# Scrub typhus as a rare cause of acute pyelonephritis: case report

**DOI:** 10.1186/s12879-020-05050-2

**Published:** 2020-05-06

**Authors:** Tulsi Bhattarai, Sujan Chandra Poudel, Nishma Pokharel, Suraj Bhattarai

**Affiliations:** 1 Department of Internal Medicine, Kathmandu Medical College, Kathmandu, Nepal; 2 Kathmandu Medical College, Kathmandu, Nepal; 3 Global Institute for Interdisciplinary Studies/Gandaki Medical College Teaching Hospital, Post Box: 24560, Sundhara, Kathmandu, Nepal

**Keywords:** Scrub typhus, Acute pyelonephritis, Urinary tract infection, Case report

## Abstract

**Background:**

Scrub typhus can present with atypical signs and symptoms such as those of acute kidney injury, gastroenteritis, pneumonitis, and acute respiratory distress syndrome. Meningitis, encephalitis, and hepatic dysfunction have also been reported, particularly in severe cases with multisystem involvement. Scrub typhus has never been reported in the literature to cause urinary tract infections (UTIs) which includes cystitis and pyelonephritis.

**Case presentation:**

A 45-year old male presenting to the outpatient unit with fever, right flank pain, and burning micturition for three days was initially treated for UTI. However, he returned to the hospital on the fourth day of illness with persistent symptoms. He was hospitalized, with intravenous (IV) ceftriaxone. Computerized tomography scan of his abdomen-pelvis showed features of acute pyelonephritis, so his antibiotics were upgraded to meropenem and teicoplanin. Despite this, the patient’s condition deteriorated. Laboratory investigations showed multisystem involvement: decreasing platelets, raised creatinine, and deranged liver panel. As Kathmandu was hit by dengue epidemic during the patient’s hospitalization, on the seventh day of his illness, blood samples were sent for tropical fever investigation. All tests came out negative except for scrub typhus—IgM antibodies positive on rapid diagnostic test. The patient’s symptoms subsided after 48 h of starting doxycycline and he became fully asymptomatic four days later. Fever did not recur even after discontinuing other IV antibiotics, favoring scrub typhus disease rather than systemic bacterial sepsis.

**Conclusions:**

Scrub typhus is an emerging infectious disease of Nepal. Therefore, every unexplained fever cases (irrespective of clinical presentation) should be evaluated for potential Rickettsiosis. Moreover, for cases with acute pyelonephritis, atypical causative agents should be investigated, for example scrub typhus in this case.

## Background

Scrub typhus is a mite-borne infectious disease caused by a bacteria called *Orientia tsutsugamushi*, previously known as *Rickettsia tsutsugamushi* [1]. It is one of the emerging infectious diseases of Nepal [2]. Clinical manifestation of scrub typhus includes a painless papule called *eschar* representing localized cutaneous necrosis at the site of infecting chigger bite, followed by fever, generalized headache, diffuse myalgia, anorexia, generalized lymphadenopathy, and non-pruritic body rash. However, it can present with atypical signs and symptoms such as those of acute kidney injury, gastroenteritis, rarely pneumonitis, and acute respiratory distress syndrome. Meningitis, encephalitis, and hepatic dysfunction have been reported too, particularly in severe cases, with multisystem involvement [3]. Case fatality rate of scrub typhus is 6% for untreated and 1.4% for treated cases [1].^,^ [4] Therefore, a high degree of clinical suspicion is required for the diagnosis of scrub typhus which can be confirmed by a rapid diagnostic test or polymerase chain reaction (PCR); Indirect immunofluorescence assay (IFA) being the gold standard test—a four-fold rise in IgM antibody titer is usually diagnostic of infection [5]. Unfortunately, in low-resource settings, it may take several weeks just to get the test results. Scrub typhus is commonly treated with doxycycline, a highly effective antibiotic given for 1 week.

Scrub typhus has never been reported in the literature to cause urinary tract infections (UTIs) which includes cystitis (infection of the bladder or lower urinary tract) and pyelonephritis (infection of the kidney or upper urinary tract). UTI is usually caused by *Escherichia coli* and rarely by other uropathogens such as other Enterobacteriaceae (*Klebsiella* spp. and *Proteus* spp.*)*, *Pseudomonas*, enterococci, and staphylococci [6]. Generally, the patients with UTI present with fever and occasionally with chills or rigors, fatigue or malaise, flank pain, costovertebral angle tenderness, and pelvic or perineal pain. Pyelonephritis can develop if pathogens ascend to the kidneys either via ureters or through the lymphatics [7].

## Case presentation

A 45 year-old male patient presented to the outpatient unit of Internal Medicine Department in September 2019 with complaints of fever, abdominal pain (right flank), and burning micturition for 3 days. He was otherwise well in the past and none of his family members had similar illness. On physical examination, he had normal temperature, blood pressure, pulse rate, and respiratory rate. On systemic examination, he had tenderness at his right renal angle. Respiratory, cardiovascular, and neurological examinations were unremarkable. There were no rash, lymphadenopathy or hepatosplenomegaly.

On urine investigation, 1–2 pus cells were seen per high power field (hpf) but no red blood cells (RBC). Total white cell count (TC) including differential counts (DC), hemoglobin (Hb), platelet count, and erythrocyte sedimentation rate (ESR) were within normal limits. His blood creatinine level was 0.9 mg/dL, urea was 33 mg/dL, sodium 142 mmol/L and potassium 4.8 mmol/L. Urine and blood culture was ordered. (Table 1*)* Ultrasonography (US) of his abdomen-pelvis showed mild fatty changes in liver, right renal concretions, and prostatomegaly (approx. 26.59 g). The patient was sent home on oral cefixime 400 mg twice daily, oral diclofenac 75 mg thrice daily, and hyoscine tablet 20 mg thrice daily.

**Table 1:** Laboratory investigation timeline and reports (abnormal values are marked in bold)

Laboratory parameters	Normal values (KMC Lab protocol)	Day 1	Day 4	Day 5	Day 6	Day 7 *(Doxycycline added)*	Day 8	Day 10	Day 17
**Hematology**									
TC (cells/mm^3^)	3500-10,500	**10,700**	**14,800**	**12,200**	**11,100**	**11,400**	**12,500**	**12,400**	7400
DC (%)	N60–80, L20–30, E1–4	**N82** **L13**	N66 L24 **E10**	**N83 L15**	**N87** **L12**	N80 **L17**	N71 L27	N73 L22	N67 L26
Hb (gm/dL)	12–16	13.4	13.7	**11.0**	**10.8**	**10.4**	**11.2**	**10.7**	**11.7**
Platelets (cells/mm^3^)	150,000-450,000	192,000	190,000	**115,000**	**120,000**	**145,000**	212,000	232,000	490,000
ESR (mm/hr)	< 15	**41**							
**Renal panel**									
Urea (mg/dL)	15–45	29	30	25	22	29	18	33	33
Creatinine (mg/dL)	0.5–1.2	1.0	**1.5**	**1.3**	1.2	1.2	1.0	1.0	0.9
Sodium (mmol/L)	135–145	138	135	136	136	136	138	138	142
Potassium (mmol/L)	3.5–5.0	3.7	3.8	3.8	**3.4**	**3.1**	3.5	3.5	4.8
**Liver panel**									
Total Bilirubin mg/dL)	0.2–1.2		**1.7**						
Direct Bilirubin (mg/dL)	0–0.4		**1.0**						
SGPT/SGOT (IU/L)	< 45 each		32/16						
ALP (IU/L)	50–100		**521**						
Albumin (gm/dL)	3.5–5.0								
PT/INR (sec)	11.0–13.5/ 0.8–1.2					**15.0/1.17**			
**Urine examination**									
Urine pus cells (per hpf)	< 1–2	**10–12**	**plenty**				0–2		
Urine RBC		nil	nil				nil		
Urine albumin		nil	nil				nil		
Urine culture		no growth	no growth		no growth				
**Other blood tests**									
Random sugar (mg/dL)	60–140	99					81		
Serum Lipase/Amylase (U/L)	10–160/ 25–110		15/25						
Blood culture		no growth							
**Tropical panel serology**									
Scrub typhus						**positive (rapid IgM)**			
Leptospira						negative			
Dengue						negative			
Leishmaniasis						negative			
Malaria (optimal test)						negative			

On Day 4, the patient returned to the outpatient unit with persistent symptoms. He was then admitted to the medical ward with intravenous (IV) ceftriaxone 1 g twice daily, Injection tramadol for pain, and intravenous fluids. Urine and blood culture reports showed no growth of pathogens. Routine laboratory investigations were repeated. Urine showed plenty pus cells but no RBCs, sugar and albumin. Serum creatinine level increased to 1.5 mg/dL whereas blood urea (30 mg/dL), sodium (135 mmol/L), and potassium (3.8 mmol/L) levels decreased. Liver panel (transaminases, total and direct bilirubin, alkaline phosphate, serum lipase, serum amylase) was normal. (Table 1*).*

The patient continued to be symptomatic on Day 5 of illness despite IV medication. Routine urine and blood investigations came out unremarkable except for a sudden decrease in platelet count (190,00 on Day 4 to 115,000 on Day 5) and serum creatinine level (1.5 on Day 4 to 1.3 on Day 5). (Table 1*)* Follow-up US abdomen-pelvis showed globular right kidney with probe tenderness, suggestive of acute pyelonephritis. Antibiotics were then upgraded to IV meropenem and IV teicoplanin.

**Fig. 1:**
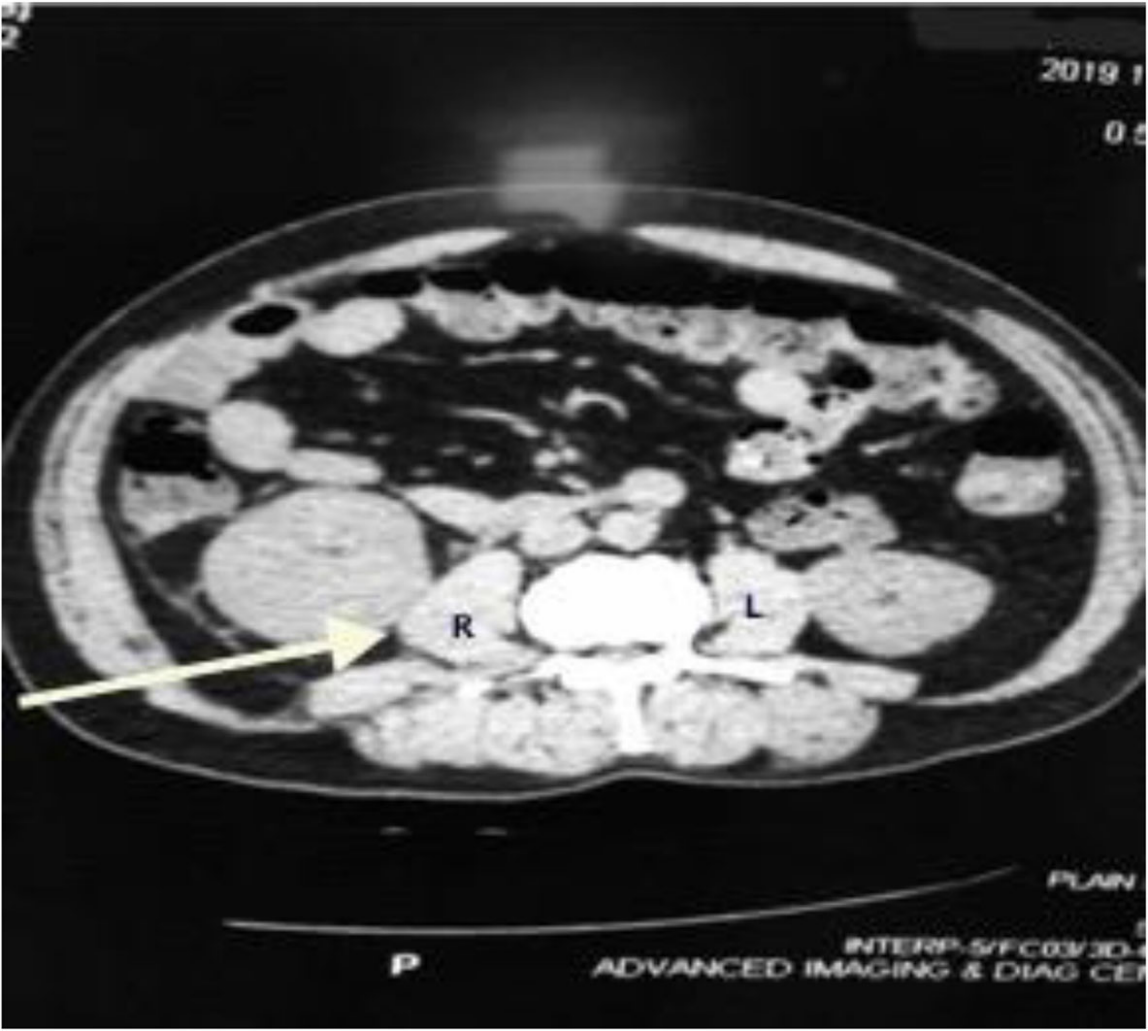
CT scan kidneys-ureters-bladder findings (on Day 5 of illness). Right kidney (R) has perirenal haziness and fatty stranding (arrow); thickened right lateral conal fascia with minimal surrounding haziness; tiny renal concretions, spenunculus, and plate atelectasis in the postero-basal segment of right lower lobe; mild degenerative changes visualized in spine. Left kidney (L) is normal

A plain computer tomography scan of the patient’s kidneys-ureters-bladder (CT-KUB) showed right perirenal haziness and fatty strandings; thickened right lateral conal fascia with minimal surrounding haziness but no evidence of hydroureteronephrosis; tiny renal concretions, splenunculus, and plate atelectasis in the posterobasal segment of right lower lobe of right kidney; and mild degenerative changes in the visualized spine. These findings complemented the US diagnosis of acute pyelonephritis. *(*Fig. 1*).*

On Day 7 of illness, the patient was still complaining right flank pain along with fever. He suddenly became tachypneic with respiratory rate of 24/min. An urgent US chest was performed which showed minimal bilateral pleural effusion. Routine laboratory investigations came out unremarkable except for decreasing creatinine level (1.2 mg/dL). (Table 1*)* Then, a possibility of serositis was suspected.

Kathmandu city was hit by dengue epidemic at the time of the patient’s hospital admission. Therefore, a possibility of tropical fever in this patient was thought of too. His blood samples were sent for the investigation for dengue virus, scrub typhus, leptospirosis, leishmaniasis (kala-azar), and malaria (optimal test). All tests came out negative except for scrub typhus –IgM antibodies positive on rapid diagnostic test. Immediately, doxycycline (100 mg IV twice daily) was added to the patient’s medication list (Day 7 of illness).

The patient’s clinical features and lab results did not change remarkably for 36 h of initiating doxycycline. However, over the next 48 h (Day 10 onwards), the patient showed clinical improvement. His fever and abdominal pain decreased significantly. On Day 12 of illness (9th day of admission, 7th day of IV meropenem, 5th day of IV doxycycline), the patient had a feeling of well-being, so he was sent home with oral doxycycline for 10 additional days and oral levofloxacin for 7 days.

When the patient visited hospital after 6 days of discharge (Day 17), he was found apparently asymptomatic; all blood and urine investigations came out normal; and his follow-up ultrasonography findings (chest-abdomen-pelvis) were non-significant.

## Discussion and conclusions

Renal abnormalities in scrub typhus case range from proteinuria or hematuria to acute kidney injury and occasionally chronic kidney disease [8]. Acute pyelonephritis in scrub typhus has been reported only once in the literature—in a 56-year-old Chinese lady who presented with frequent micturition, flank pain, and an *eschar* in her body [9]. The mechanisms postulated for renal involvement include typhus-related vasculitis, tubular interstitial proliferation, and tubular necrosis [8].

In the current case, poor control of symptoms with oral and intravenous antibiotics (cefixime, ceftriaxone, meropenem and teicoplanin), abnormal laboratory and radiographic reports suggestive of multiorgan involvement (decreasing platelet counts, decreasing creatinine, deranged liver panel, globular right kidney, bilateral pleural effusion), and an ongoing tropical fever epidemic in Kathmandu prompted the clinicians to investigate for an atypical causative agent.

Positive rapid serological test (IgM antibodies against scrub typhus) confirmed the diagnosis. All investigation reports including US chest-abdomen-pelvis drastically came out normal, with no evidence of pyelonephritis, after 10 days of initiating doxycycline (Day 17 of onset of illness). Moreover, patient’s fever did not recur even after discontinuing other intravenous antibiotics, favoring scrub typhus diseaseccc rather than systemic bacterial sepsis.

Acute febrile illness can present with atypical clinical signs and symptoms, often with multisystem involvement, that should urge clinicians to look for atypical pathogens—for example scrub typhus associated with pyelonephritis in this case. Scrub typhus is a common but neglected tropical disease in South Asia including Nepal. Therefore, stakeholders should not wait until an outbreak or epidemic occurs to initiate standard surveillance programs, deploy reliable and affordable diagnostic kits at all levels of healthcare service, and raise community awareness about disease transmission and preventive measures.

## Data Availability

All the information supporting our conclusions and relevant references are included in the manuscript. There are no datasets related to this case report.

## Abbreviations

ALP: Alkaline phosphatase
CT: Computerized tomography
DC: Differential count
ESR: Erythrocyte sedimentation rate
Hb: Hemoglobin
IFA: Immunofluorescence assay
KMC: Kathmandu medical college
KUB: Kidneys-ureters-bladder
PCR: Polymerase chain reaction
PT/INR: Prothrombin time/International normalized ratio
SGPT/SGOT: Serum glutamic pyruvic transaminase/oxaloacetic transaminase
TLC: Total leucocyte count
UTI: Urinary tract infection
